# Fusobacterium necrophorum Brain Abscess With Concurrent Nasopharyngeal Detection of Mycoplasma pneumoniae: A Case Report

**DOI:** 10.7759/cureus.108808

**Published:** 2026-05-13

**Authors:** Zehdi Eydou, Scott Garber, Megan Sikkema

**Affiliations:** 1 Department of Family and Community Medicine, Western Michigan University Homer Stryker M.D. School of Medicine, Kalamazoo, USA; 2 Department of Pediatric and Adolescent Medicine, Western Michigan University Homer Stryker M.D. School of Medicine, Kalamazoo, USA; 3 Department of General Pediatrics, Bronson Children's Hospital, Kalamazoo, USA

**Keywords:** brain abscess, central nervous system infection, fusobacterium necrophorum, lemierre syndrome, mycoplasma pneumoniae, polymerase chain reaction

## Abstract

*Fusobacterium necrophorum* is a recognized cause of brain abscesses and has been classically associated with Lemierre syndrome. We report a case of a 17-year-old male patient who presented with seizures, fever, and altered mental status following treatment for sinusitis. Neuroimaging revealed a right frontal lobe abscess requiring surgical drainage. Anaerobic cultures from the abscess fluid grew* F. necrophorum*, while a respiratory polymerase chain reaction (PCR) panel from a nasopharyngeal swab detected* Mycoplasma pneumoniae*. Anaerobic cultures also yielded *Paenibacillus barengoltzii* from a separate soft tissue specimen, though its clinical significance remained uncertain. The patient improved with surgical drainage and targeted antimicrobial therapy. This case highlights concurrent detection of *F. necrophorum* and *M. pneumoniae* in a central nervous system infection and underscores the uncertainty regarding the pathogenic role of *M. pneumoniae* in such presentations.

## Introduction

*Fusobacterium necrophorum* is classically associated with Lemierre syndrome, a rare septic thrombophlebitis of the internal jugular vein that typically follows oropharyngeal infection and may result in metastatic complications [1]. The central nervous system involvement due to* F. necrophorum* most commonly occurs through a contiguous spread from head and neck infections such as sinusitis or otitis media [1,2]. Respiratory polymerase chain reaction (PCR) detection of *Mycoplasma pneumoniae* in patients with focal intracranial infections presents a diagnostic challenge, as positive nasopharyngeal testing may reflect active infection, asymptomatic carriage, or incidental upper respiratory tract detection [3,4]. Reports describing concurrent detection of *M. pneumoniae *in invasive *F. necrophorum* infections remain limited [5-8]. This case highlights the diagnostic uncertainty surrounding the interpretation of respiratory PCR findings in polymicrobial central nervous system (CNS) infections.

## Case presentation

Presentation

A 17-year-old male patient presented to the emergency department with his mother for fevers, seizures, and altered mental status. Prior to this presentation, he had rhinorrhea, congestion, and frontal headache for about three weeks. He was prescribed oral amoxicillin-clavulanate 875-125 mg twice daily for 10 days from an outpatient visit to treat acute bacterial sinusitis. His headache progressively worsened during the final days of antibiotic therapy and continued after completion of the 10-day course. Also, he developed intermittent fevers for several days as high as 102.4°F (39°C) when the headache worsened. Two days after completing therapy, he developed generalized tonic-clonic seizures, prompting an emergency evaluation. He was given intramuscular midazolam 5 mg once by emergency medical services while being transferred to the hospital. He had a history of depression, which was well-controlled on oral bupropion 300 mg daily. He had no significant past surgical history.

On arrival at the hospital, he was unable to provide his medical history and had a significantly depressed mental status. He was responsive only to painful stimuli. On physical examination, he was ill-appearing with an altered mental status. His pupils were markedly dilated bilaterally and sluggish. His Glasgow Coma Scale was 5/15 with an eye subscore of 1/4, a verbal subscore of 1/5, and a motor subscore of 5/6. He was intubated and admitted to the pediatric intensive care unit.

Investigations

**Figure 1:**
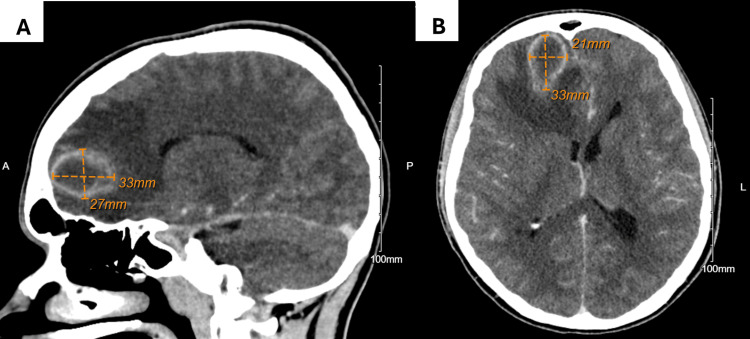
CT scan of the brain demonstrating the right frontal lobe abscess The sagittal view (A) and axial view (B) of the brain CT scan with and without contrast on admission, revealing a 3.3 cm x 2.1 cm x 2.7 cm right frontal lobe abscess.

A complete blood count revealed a white blood cell count of 19.4x10^9^/L. A brain CT scan showed a 3.3 cmx2.1 cmx2.7 cm right frontal lobe abscess with surrounding vasogenic edema (Figure 1). A respiratory PCR panel from a nasopharyngeal swab detected *M. pneumoniae* on day one of hospitalization. A stereotactic bilateral frontal craniotomy with irrigation was performed by neurosurgery to drain the right frontal abscess fluid. Intraoperative findings included frank purulence within both the abscess cavity and the frontal sinus, consistent with concurrent sinusitis. Approximately 8 mL of purulent material was drained.

**Figure 2:**
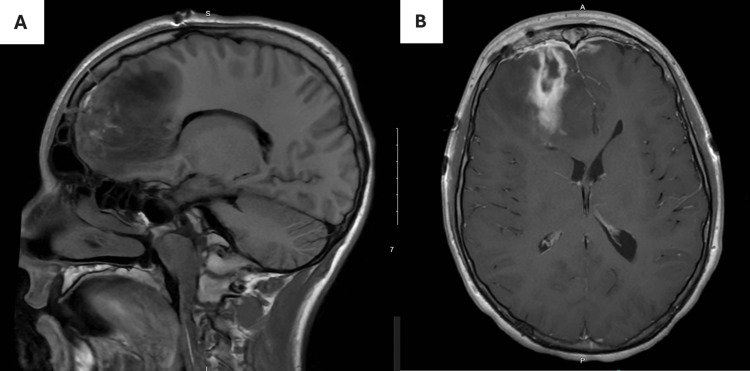
Postoperative MRI of the brain demonstrating residual edema and midline shift The sagittal view (A) and axial view (B) of the brain T1-weighted MRI with contrast six days postoperatively, revealing post-surgical changes, including persistent vasogenic edema with a 4 mm right-to-left midline shift.

Intraoperative specimens from the abscess and frontal sinus were sent for aerobic, anaerobic, and fungal cultures. Anaerobic culture from abscess fluid yielded *F. necrophorum*. A separate anaerobic soft tissue culture yielded *Paenibacillus barengoltzii*. Additional anaerobic cultures showed no growth. Aerobic bacterial and fungal cultures demonstrated no growth. Gram stains from abscess fluid and soft tissue samples showed numerous white blood cells without identifiable organisms. Blood cultures obtained on admission (two sets) were negative. No repeat blood cultures were obtained. A brain MRI after surgery demonstrated postoperative changes with persistent vasogenic edema and mild midline shift (Figure 2).

The differential diagnosis included subdural empyema, meningitis, and cerebral venous sinus thrombosis. However, imaging findings and microbiological cultures supported the diagnosis of a bacterial brain abscess, likely secondary to contiguous spread from frontal sinusitis. There was no clinical or radiographic evidence of internal jugular vein thrombosis or dural venous sinus thrombosis, and Lemierre syndrome was not clinically suspected. Hence, dedicated venous imaging was not performed. Lumbar puncture and cerebrospinal fluid analysis were not performed because the presence of a large intracranial abscess with mass effect and midline shift raised concern for increased intracranial pressure and potential herniation risk. The etiology of the dilated and sluggish pupillary findings was uncertain and may have reflected recent benzodiazepine administration, elevated intracranial pressure, or both.

Treatment

On admission, he was started on broad-spectrum antibiotics with intravenous vancomycin 1 g every eight hours, intravenous metronidazole 500 mg every six hours, and intravenous cefepime 2 g every eight hours. After his surgery, the patient was successfully extubated to room air. After extubation, he was oriented to time, place, and person and was able to follow commands. He was initially continued on broad-spectrum antibiotics, and oral azithromycin was added with a loading dose of 500 mg once, followed by 5 mg/kg daily for a total of five days.

Based on the culture results confirming *F. necrophorum*, antimicrobial therapy was narrowed to oral metronidazole 500 mg every eight hours and intravenous ceftriaxone every 12 hours after receiving four days of broad-spectrum antibiotics. Simultaneously, the five-day course of azithromycin was completed to address possible *M. pneumoniae *infection. Because *M. pneumoniae* was detected only on nasopharyngeal PCR without direct evidence of CNS involvement, standard-duration azithromycin therapy for respiratory infection was considered appropriate. Also, oral levetiracetam 1 g twice daily was started for seizure prophylaxis for a total of seven days.

Follow-up

**Figure 3:**
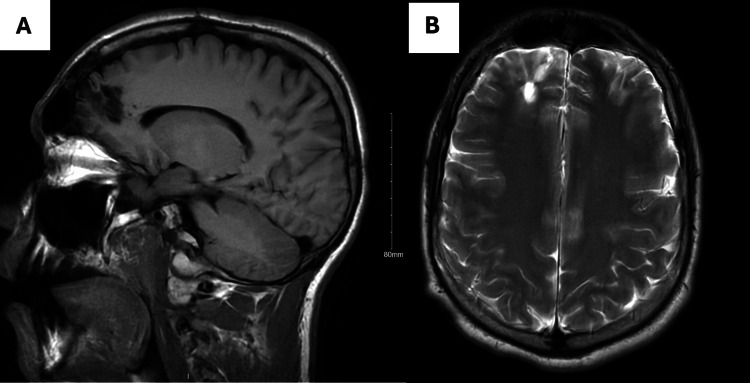
Postoperative MRI of the brain at six-month follow-up The sagittal view (A) and axial view (B) of the T2-weighted magnetic resonance imaging with contrast six months postoperatively, revealing postsurgical changes at the site of the right frontal lobe craniotomy with encephalomalacia.

The patient was discharged to an inpatient rehabilitation facility and recovered without residual neurological deficits. He was continued on the ceftriaxone and metronidazole on an outpatient basis until he completed a total of eight weeks of antibiotic treatment. Over the year following discharge, the patient experienced multiple readmissions for seizure recurrence; however, there was no evidence of recurrent infection. Follow-up neuroimaging demonstrated only postsurgical changes without new abscess formation (Figure 3). Antiseizure therapy was resumed and adjusted with subsequent clinical stabilization. The seizure recurrences were attributed to medication nonadherence rather than ongoing infection.

## Discussion

*F. necrophorum* is an established cause of brain abscesses and is classically associated with Lemierre syndrome [1]. Its role in invasive head and neck infections with intracranial extension is well-described [2]. In the present case, *F. necrophorum* was isolated from abscess fluid cultures, confirming it as the primary etiologic agent of the brain abscess. The presence of concurrent frontal sinusitis supports contiguous spread as the most likely source of infection. *M. pneumoniae* was detected on a PCR panel obtained early in the clinical course. There was no clinical or radiological evidence of Lemierre syndrome. In addition to* F. necrophorum*, anaerobic cultures yielded *P. barengoltzii *from a separate soft tissue specimen.

Several reports described concurrent detection of *M. pneumoniae* in cases of *F. necrophorum*-associated Lemierre syndrome (Table 1). Moreover, *M. pneumoniae* was implicated as the sole cause of a brain abscess (Table 2) and encephalitis [9]. Together, these observations suggest that* M. pneumoniae* may be detected in complex infections involving the CNS, although its role as a true pathogen in such settings remains uncertain [3]. Details of the literature search strategy are provided in the Appendices. Compared with several previously reported cases involving neurological deficits, recurrent meningitis, or prolonged complications, our patient experienced a favorable neurological recovery following prompt surgical drainage and targeted antimicrobial therapy. 

**Table 1:** Reported cases of Lemierre syndrome due to Fusobacterium necrophorum with concurrent detection of Mycoplasma pneumoniae An excerpt of four cases of Lemierre syndrome due to *F. necrophorum*, in which *M. pneumoniae *was identified. CFA: complement fixation assay. CT: computed tomography. ELISA: enzyme-linked immunosorbent assay. IJV: internal jugular vein. MAA: microparticle agglutination assay. MRI: magnetic resonance imaging. PCR: polymerase chain reaction. PMH: past medical history. SN: serial number. Y/O: year-old.

SN	Study	Presentation	Radiology and Labs	Microbiology	Treatment	Follow Up
1	Walizada et al. [5]: case report	Male, 24 Y/O, no significant PMH: presented with shortness of breath and pleuritic chest pain	Initial chest CT showed bilateral pulmonary nodules; neck CT showed a right peritonsillar abscess; repeat chest CT showed stable pulmonary nodules with a new right lower lobe consolidation and worsening pleural effusion	Initial blood cultures grew *F. necrophorum*; positive IgM to *M. pneumoniae *(low positive range)	Metronidazole, ampicillin/sulbactam, dexamethasone, incision and drainage of the peritonsillar abscess, and right-sided thoracentesis	Clinically improved and discharged; continued ampicillin/sulbactam for 10 days after discharge, followed by two weeks of amoxicillin/clavulanate
2	Chen et al. [6]: letter to the editor	Male, 19 Y/O, PMH not provided: presented with fever, sore throat, and dyspnea for three days	Chest CT showed infiltration of the right lower lobe with pleural effusion and left IJV thrombosis; pleural fluid analysis showed an exudative effusion	Blood, pleural fluid, and middle ear pus cultures grew* F. necrophorum*; initially, positive IgM at 6.5 and positive IgG at 1280 to *Mycoplasma*; IgG decreased to 1:320 in the convalescent stage	Initially, amoxicillin/clavulanate; later, switched to levofloxacin and metronidazole to complete a total of 21 days	Not provided
3	Meis et al. [7]: case report	Female, 6 Y/O, no significant PMH: initially treated as an outpatient for otitis media; later, developed right-sided hemiplegia and homonymous hemianopsia, in addition to septic complications of the hip and big toe	Brain CT showed four ring-enhancing lesions in the left parietal and occipital lobes	Positive CFA for *M. pneumoniae *(titer increased from 1:64 to 1000:4096) with a positive IgM and IgA ELISA; the first blood culture and abscess culture grew* F. necrophorum*	Initially, erythromycin, trimethoprim/sulfamethoxazole, and bur hole aspiration of one abscess, revealing necrotic brain tissue and pus; later, switched to metronidazole and penicillin G	Discharged with right-sided hemiparesis after completing eight weeks of antibiotics; brain CT on discharge revealed a small residual parietal lesion; follow-up after 10 months revealed adequate functional recovery and total radiological resolution
4	Abele-Horn et al. [8]: case report	Male, 20 Y/O, no significant PMH: initially treated for pneumonia with clarithromycin; developed fever, dyspnea, and lower back pain three weeks later	Thoracic spine CT showed bilateral pulmonary abscesses; lumbar spine MRI showed spondylodiskitis at L4-L5	Initially, at the time of pneumonia diagnosis, positive MAA (titer 1:320), IgM and IgG in the immunoblot, and PCR for* M. pneumoniae* (specimen source not provided); later, positive oropharyngeal swabs and blood cultures for *F. necrophorum* and positive MAA (titer 1:40) and IgG response in the immunoblot to *M. pneumoniae*	Initially, ampicillin/sulbactam; later switched to imipenem due to lack of clinical response; a corset was applied for three months	Discharged after four weeks with no residual neurological sequelae; repeat CT and MRI scans after 12 months were normal

**Table 2:** A reported case of a brain abscess attributed to Mycoplasma pneumoniae An excerpt of a case of a brain abscess caused by *M. pneumoniae*. CPA: cerebellopontine angle. CSF: cerebrospinal fluid. DNA: deoxyribonucleic acid. IJV: internal jugular vein. MRI: magnetic resonance imaging. MS: multiple sclerosis. PCR: polymerase chain reaction. rDNA PCR: ribosomal DNA PCR, rtPCR: real-time PCR. SN: serial number. Y/O: year-old.

SN	Study	Presentation	Diagnosis	Microbiology	Treatment	Follow Up
1	Madžar et al. [10]: case report	Female, 21 Y/O, with a history of MS on ocrelizumab: initially treated with multiple antibiotics for bacterial meningitis; later, presented with new mild right facial palsy	Initial brain MRI showed right IJV and sigmoid sinus contrast enhancement; repeat brain MRI showed a right CPA abscess	Initially, CSF analysis was consistent with bacterial infection, although the cultures and 16S rDNA PCR were negative; repeat CSF 16S rDNA PCR resulted positive for *M. pneumoniae*, which was confirmed using specific rtPCR primers	Treated with doxycycline; repeat brain MRIs showed gradual abscess regression, and repeat CSF analysis showed improvement	Fever recurred four weeks later, and repeat CSF analysis confirmed meningitis recurrence; doxycycline was restarted and levofloxacin was added, resulting in clinical and radiological (MRI) improvement; lifetime antibiotics were then continued

The detection of *M. pneumoniae* in the present case may represent an incidental detection without pathogenic relevance, whether as an independent respiratory infection or as colonization. Alternatively, it may reflect coinfection or a preceding infection facilitating subsequent *F. necrophorum* disease (superinfection). Interpretation is limited by the absence of serological testing and the use of respiratory PCR, which may reflect asymptomatic carriage or incidental upper respiratory tract detection rather than true pathogenic involvement in the context of *Mycoplasma *spp*. *[4].

The isolation of *P. barengoltzii* from the abscess culture is of uncertain significance and further complicates interpretation. This organism has not previously been reported in association with human brain abscesses, and its pathogenic potential in CNS infections is unknown. Its detection may reflect improved anaerobic culture techniques or broader microbial diversity in polymicrobial brain abscesses than is currently recognized. Whether* P. barengoltzii* represents contamination, transient colonization, or a contributory pathogen cannot be definitively determined from this case.

## Conclusions

In conclusion, while *F. necrophorum* was the confirmed cause of the brain abscess in the present case, the concurrent detection of *M. pneumoniae* highlights an infrequently described finding that warrants further investigation. Clarifying whether this finding represents incidental detection, coinfection, or superinfection may help improve our understanding of complex CNS infections and inform future diagnostic and therapeutic strategies. Future studies incorporating broader anaerobic sequencing and advanced microbiological characterization may help clarify the contribution of polymicrobial organisms in CNS abscesses. Additionally, this case highlights the importance of cautious interpretation of respiratory PCR findings in the setting of a focal intracranial infection, as detection may not necessarily reflect pathogenic involvement at the site of disease. The additional isolation of *P. barengoltzii* further highlights the potential microbiological complexity of polymicrobial CNS infections.

## Appendices

Literature review

A literature review was conducted to examine the incidence of brain abscesses associated with an infection with one of the following three organisms: *M. pneumoniae*,* F. necrophorum*, and *P. barengoltzii*. In addition, the literature review was expanded to include results that mentioned any two of the above three organisms to search for reports of combined infections by those microorganisms that did not involve the brain. PubMed was searched for articles published up to January 1, 2026, with the following search string:

(("brain abscess"[MeSH Terms] OR ("brain"[All Fields] AND "abscess"[All Fields]) OR "brain abscess"[All Fields] OR ("brain abscess"[MeSH Terms] OR ("brain"[All Fields] AND "abscess"[All Fields]) OR "brain abscess"[All Fields] OR ("cerebral"[All Fields] AND "abscess"[All Fields]) OR "cerebral abscess"[All Fields]) OR (("cerebellum"[MeSH Terms] OR "cerebellum"[All Fields] OR "cerebellar"[All Fields]) AND ("abscess"[MeSH Terms] OR "abscess"[All Fields] OR "abscesses"[All Fields] OR "abscessation"[All Fields] OR "abscessed"[All Fields] OR "abscessing"[All Fields])) OR (("brain stem"[MeSH Terms] OR ("brain"[All Fields] AND "stem"[All Fields]) OR "brain stem"[All Fields] OR "brainstem"[All Fields] OR "brainstems"[All Fields] OR "brainstem s"[All Fields]) AND ("abscess"[MeSH Terms] OR "abscess"[All Fields] OR "abscesses"[All Fields] OR "abscessation"[All Fields] OR "abscessed"[All Fields] OR "abscessing"[All Fields])) OR (("brain stem"[MeSH Terms] OR ("brain"[All Fields] AND "stem"[All Fields]) OR "brain stem"[All Fields]) AND ("abscess"[MeSH Terms] OR "abscess"[All Fields] OR "abscesses"[All Fields] OR "abscessation"[All Fields] OR "abscessed"[All Fields] OR "abscessing"[All Fields]))) AND ("mycoplasma pneumoniae"[MeSH Terms] OR ("mycoplasma"[All Fields] AND "pneumoniae"[All Fields]) OR "mycoplasma pneumoniae"[All Fields] OR ("paenibacillus barengoltzii"[Supplementary Concept] OR "paenibacillus barengoltzii"[All Fields] OR "paenibacillus barengoltzii"[All Fields]) OR ("fusobacterium necrophorum"[MeSH Terms] OR ("fusobacterium"[All Fields] AND "necrophorum"[All Fields]) OR "fusobacterium necrophorum"[All Fields]))) OR (("mycoplasma pneumoniae"[MeSH Terms] OR ("mycoplasma"[All Fields] AND "pneumoniae"[All Fields]) OR "mycoplasma pneumoniae"[All Fields]) AND ("fusobacterium necrophorum"[MeSH Terms] OR ("fusobacterium"[All Fields] AND "necrophorum"[All Fields]) OR "fusobacterium necrophorum"[All Fields])) OR (("mycoplasma pneumoniae"[MeSH Terms] OR ("mycoplasma"[All Fields] AND "pneumoniae"[All Fields]) OR "mycoplasma pneumoniae"[All Fields]) AND ("paenibacillus barengoltzii"[Supplementary Concept] OR "paenibacillus barengoltzii"[All Fields] OR "paenibacillus barengoltzii"[All Fields])) OR (("fusobacterium necrophorum"[MeSH Terms] OR ("fusobacterium"[All Fields] AND "necrophorum"[All Fields]) OR "fusobacterium necrophorum"[All Fields]) AND ("paenibacillus barengoltzii"[Supplementary Concept] OR "paenibacillus barengoltzii"[All Fields] OR "paenibacillus barengoltzii"[All Fields]))

Only English-language and translated articles were reviewed. The search yielded 57 results with no duplicates. After screening the titles and abstracts, 18 results were excluded, leaving 39 for full-text review. Upon full-text review, 27 articles were excluded due to the absence of *M. pneumoniae* and *P. barengoltzii *involvement in abscesses, Lemierre disease, and/or infections involving *F. necrophorum* and other microorganisms. Five articles were excluded because their full texts were not available in the English language. One article was excluded because its full text was not available. One article was excluded because of no involvement of *F. necrophorum* and *P. barengoltzii* in a case of encephalitis due to *M. pneumoniae *with no abscess formation. The remaining five articles were included, as summarized in Tables 1, 2 in the manuscript.
